# High-sensitivity cardiac troponin T is a biomarker for atherosclerosis in systemic lupus erythematous patients: a cross-sectional controlled study

**DOI:** 10.1186/s13075-017-1352-7

**Published:** 2017-06-13

**Authors:** Gillian Divard, Rachid Abbas, Camille Chenevier-Gobeaux, Noémie Chanson, Brigitte Escoubet, Marie-Paule Chauveheid, Antoine Dossier, Thomas Papo, Monique Dehoux, Karim Sacre

**Affiliations:** 1 Département de Médecine Interne, Hôpital Bichat, Université Paris Diderot, PRES Sorbonne Paris Cité, Assistance Publique Hôpitaux de Paris, 46 rue Henri Huchard, 75018 Paris, France; 2 Département d’Epidémiologie et recherche clinique, CIC-EC 1425, Groupe Hospitalier Paris Nord Val de Seine, AP-HP, Hôpital Bichat, Paris France; Univ Paris Diderot, PRES Sorbonne Paris Cité, UMR 1123 ECEVE, Paris, France; 3 Département de Biochimie, Hôpital Cochin, Université Paris Descartes, PRES Sorbonne Paris Cité, Assistance Publique Hôpitaux de Paris, Paris, France; 4 Département de Physiologie, Hôpital Bichat, Université Paris Diderot, PRES Sorbonne Paris Cité, Assistance Publique Hôpitaux de Paris, INSERM U1138, Paris, France; 5 INSERM U1149, Paris, France; 6 Département Hospitalo-Universitaire FIRE (Fibrosis, Inflammation and Remodelling in Renal and Respiratory Diseases), Paris, France; 7 Département de Biochimie Métabolique et Cellulaire, Hôpital Bichat, Université Paris Diderot, PRES Sorbonne Paris Cité, Assistance Publique Hôpitaux de Paris, Paris, France

**Keywords:** Lupus, Atherosclerosis, Biomarker

## Abstract

**Background:**

Cardiovascular disease (CVD) is the main cause of death in systemic lupus erythematous (SLE) patients. The Framingham score underestimates the risk for CVD in this population. Our study aimed to determine whether serum high-sensitivity cardiac troponin T (HS-cTnT) might help to identify SLE patients at risk for CVD.

**Methods:**

The presence of carotid plaques was prospectively assessed by ultrasound in 63 consecutive SLE patients asymptomatic for CVD and 18 controls. Serum HS-cTnT concentration was measured using the electrochemiluminescence method. Factors associated with carotid plaques were identified and multivariate analysis was performed.

**Results:**

Framingham score was low in both SLE patients (median 1 (range 1–18%)) and controls (1 (1–13%)). Nevertheless, 23 (36.5%) SLE patients, but only 2 (11.1%) controls (*p* = 0.039), had carotid plaque detected by vascular ultrasound. In the multivariate analysis, only age (*p* = 0.006) and SLE status (*p* = 0.017) were independently associated with carotid plaques. Serum HS-cTnT concentration was detectable (i.e. >3 ng/L) in 37 (58.7%) SLE patients and 6 (33.3%) controls (*p* = 0.057). Interestingly, 87% of SLE patients with carotid plaques, but only 42.5% of SLE patients without plaques (*p* < 0.001), had detectable HS-cTnT. Conversely, 54.5% of SLE patients with detectable HS-cTnT, but only 11.5% with undetectable HS-cTnT (*p* < 0.001), had a carotid plaque. In the multivariate analysis, only body mass index (*p* = 0.006) and HS-cTnT (*p* = 0.033) were statistically associated with carotid plaques in SLE patients. Overall, the risk of having a carotid plaque was increased by 9 (odds ratio 9.26, 95% confidence interval 1.55–90.07) in SLE patients in whom HS-cTnT was detectable in serum.

**Conclusion:**

Serum HS-cTnT level is high and associated with carotid plaques in SLE patients who are at an apparently low risk for CVD according to the Framingham score. HS-cTnT may be a useful biomarker for SLE-associated atherosclerosis.

## Background

Cardiovascular disease (CVD) is now recognized as the leading cause of death in systemic lupus erythematosus (SLE) patients [1]. Although traditional cardiovascular risk factors contribute to early-onset atherosclerosis in SLE, the phenomenon is not fully explained by a higher frequency of smoking habits, hypertension, or dyslipidaemia in this population [2–5]. Accordingly, the Framingham risk equation usually underestimates the 10-year cardiovascular risk in the SLE population [6]. Thus, identification of biological markers able to better stratify cardiovascular risks in SLE patients is needed.

Cardiac troponin (cTnT) is a well-known marker of myocyte necrosis and injury in the early phase of acute myocardial infarction [7, 8]. Measured with high-sensitivity (HS) assays, HS-cTnT has a proven predictive value for coronary heart disease, heart failure, and mortality in the general population at apparent low-risk for CVD [9]. There are, however, no data regarding the predictive value of HS-cTnT in the context of SLE.

The aim of this study was to determine whether HS-cTnT was associated with accelerated atherosclerosis established by carotid ultrasonography in SLE patients who are at an apparently low risk for CVD according to traditional risk factors.

## Methods

### Study participants

Sixty-three consecutive patients with SLE followed in the Department of Internal Medicine, Bichat Hospital, Paris-Diderot University, Paris, were enrolled between January 2012 and January 2013. All subjects fulfilled at least four of the American College of Rheumatology criteria for SLE [10]. Exclusion criteria consisted of known coronary disease or symptoms suggestive of CVD (angina, arrhythmia, congestive heart failure, stroke, and peripheral arterial disease). Controls were volunteer health workers who had prospectively undergone vascular ultrasound imaging during the same period of time. None had coronary disease or symptoms suggestive of CVD. The first patient with SLE was considered with respect to age and sex. The health worker group was examined to find the first control patient of the same age ±3 years and sex. Matches within the range were found for 18 SLE participants. The risk for cardiovascular events was calculated as the absolute risk within the next 10 years using the Framingham risk equation, which includes age, sex, total cholesterol level, high-density lipoprotein cholesterol level, smoking history, and systolic blood pressure. Subjects were considered to have hypertension if they repeatedly had a systolic blood pressure of at least 140 mm Hg or a diastolic blood pressure of at least 90 mm Hg. Height and weight were measured, and the body mass index (BMI) was calculated as the weight in kilograms divided by the square of the height in meters. SLE disease activity was assessed using the Safety of Estrogens in Lupus Erythematosus National Assessment (SELENA)-Systemic Lupus Erythematosus Disease Activity Index (SLEDAI) score [11]. The diagnosis of antiphospholipid syndrome (APS) was based on a history of venous and/or arterial thromboses or recurrent miscarriages in the presence of aPL antibodies in accordance with published criteria [12]. Lupus nephritis diagnosis was based on International Society of Nephrology/Renal Pathology Society classification [13]. The local ethics committee approved the study (Institutional Review Board IRB 00006477 of HUPNVS, Paris 7 University, AP-HP). All patients provided written informed consent.

### Vascular assessment

The vascular ultrasound study was performed in the context of care in a temperature-controlled room after a 15-min rest (Vivid 7, General Electric, Horten, Norway). All subjects had fasted for at least 12 h before vascular evaluation. A single blinded investigator (BE) conducted vascular measurements in the controls and SLE patients. All data were analysed offline (EchoPAC™, General Electric Ultrasound). The internal carotid artery was imaged in a longitudinal and cross-sectional view. The maximal thickness was measured as the internal carotid wall thickness at the carotid bulb level at end diastole, as gated on electrocardiography (ECG). Carotid plaques were defined as a thickness greater than 1.5 mm according to current recommendation [14]. Intima media thickness (IMT) was measured offline from B-mode diastolic images, as gated on ECG by semiautomatic detection with dedicated software (General Electric).

### HS-cTnT measurement

HS-cTnT measurements were performed on the e602 immunomodule of the cobas 8000 analyser (Roche Diagnostics, Meylan, France) using the HS-cTnT Elecsys®2010 immunoassay. This assay is based on a one-step sandwich principle, with electrochemiluminescent revelation. The total duration of the assay is 18 min, and 50 μL of the sample is incubated with an anti-cTnT monoclonal antibody labelled with ruthenium and with a biotinylated monoclonal anti-cTnT antibody. According to the manufacturer, the measurement range of the assay is 3 to 10,000 ng/L; the 99th percentile value is 14 ng/L, and the 10% coefficient of variation (CV) value is 13 ng/L (data from the manufacturer). Our laboratory CVs during the study measurements were 2.6% (at 29.6 ng/L) and 2.4% (at 2196 ng/L) as previously reported [15].

### Statistical analysis

Continuous variables are expressed as the median (range). Categorical variables are expressed as frequencies and percentages. Data were compared between SLE patients and controls using the chi-squared (or Fisher) test for categorical variables and the Mann-Whitney *U* test for continuous variables. The presence of carotid plaques (yes/no) was used as the explanatory variable. Factors identified in the univariate analysis (with a *p* value below 10%) were used in a multivariate analysis (logistic regression) to identify those independently associated with carotid plaques. The same analysis was conducted on the SLE patients, with the addition of the following SLE-specific factors: HS-cTnT, estimated glomerular filtration rate (eGFR), proteinuria/creatininuria, duration of SLE disease, SELENA-SLEDAI score, lupus nephritis, antiphospholipid (AP) antibodies, APS, cumulative years of steroid treatment, hormonal contraception, hydroxychloroquine, and immunosuppressive therapy. Before introduction in the model, interactions between explanatory variable were explored. The Akaike information criterion was used in a stepwise procedure to retain the best model. We checked the linearity for each continuous predictor using the residuals versus fitted values plot, and we used likelihood ratio test with models using non-linear transformations. Statistical analyses were performed with GraphPad Prism 5.01 software and R statistical software version 3.2 (R Foundation for statistical Computing, Vienna, Austria). All tests were two-sided and *p* values <0.05 were considered statistically significant.

## Results

### Characteristics of SLE patients and controls

Sixty-three consecutive SLE patients (39 (29–63) years old, 82.5% female gender) asymptomatic for cardiovascular disease and 18 controls (41 (30–61) years old, 77.8% female gender) were studied. The absolute risk of cardiovascular events occurring within the next 10 years according to the Framingham score was 1 (1–18)% and 1 (1–13)% in SLE patients and controls, respectively (*p* = 0.416). Age, sex, tobacco use, hypertension, waist circumference, and BMI were not statistically different between SLE patients and controls. Low density lipoprotein cholesterol level was lower in SLE patients compared to controls (0.90 (0.36–1.53) vs 1.22 (0.74–1.72) g/l, *p* = 0.001). Neither SLE patients nor controls had diabetes. Clinical characteristics of SLE patients and controls are shown in detail in Table 1.

**Table 1:** Characteristics of SLE patients and controls subjects

	SLE (*n* = 63)	Controls (*n* = 18)	*p* value
Age, years	39 (29–63)	41 (30–61)	0.357
Female gender, *n* (%)	52 (82.5)	14 (77.8)	0.646
Smoker current, *n* (%)	24 (38.1)	8 (44.4)	0.627
LDL cholesterol, g/L	0.90 (0.36–1.53)	1.22 (0.74–1.72)	0.001
Hypertension, *n* (%)	16 (25.4)	4 (22.2)	0.783
SBP, mm Hg	125 (100–166)	125 (108–184)	0.498
10-Year cardiovascular risk^a^, %	1 (1–18)	1 (1–13)	0.416
Overweight^b^, *n* (%)	24 (38.1)	8 (44.4)	0.627
Waist circumference, cm	90 (74–150)	91 (74–117)	0.852
eGFR^c^, mL/min/1.73 m^2^	92 (54–127)	76 (60–99)	0.221
HbA1c, %	5.5 (4.4–6.7)	5.5 (4.6–6.5)	0.988
Antiplatelet treatment, *n* (%)	7 (11.1)	1 (5.6)	0.486
Statin, *n* (%)	15 (23.8)	2 (11.1)	0.139
ACE inhibitors, *n* (%)	16 (25.4)	3 (16.7)	0.440
ARBs, *n* (%)	5 (7.9)	0	0.217
Carotid plaque^d^, *n* (%)	23 (36.5)	2 (11.1)	0.039

Results are shown as median (range) or number (percentage)

Analysis was performed on 81 subjects

^a^10-year cardiovascular risk was calculated using the Framingham equation

^b^Overweight was defined as a body mass index >25 kg/m^2^

^c^Estimated glomerular filtration rate (eGRF) was calculated with the Modification of Diet in Renal Disease (MDRD) equation

^d^Carotid plaques were defined as an internal carotid wall thickness at the carotid bulb >1.5 mm

*ACE* Angiotensin converting enzyme, *ARB* Angiotensin II receptor antagonist, *HbA1c* haemoglobin A1c, *LDL* low-density lipoprotein, *SBP* systolic blood pressure, *SLE* systemic lupus erythematosus

### Lupus is an independent risk factor for carotid plaque in patients at apparent low risk for cardiovascular disease

While both groups shared a low Framingham risk score, 23 (36.5%) SLE patients but only 2 controls (11.1%) had a carotid atherosclerotic plaque (i.e. a local internal carotid wall thickening >1.5 mm) identified by vascular ultrasound (*p* = 0.039). In the multivariate analysis (Table 2), only age (*p* = 0.006) and SLE status (*p* = 0.017) were independently associated with carotid atherosclerotic plaque. Of note, a status of SLE appeared to increase the risk for subclinical atherosclerosis by more than 9 (odds ratio (OR) 9.16, 95% confidence interval (CI) 1.82–77.97) in patients at apparent low risk for CVD.

**Table 2:** Multivariate analysis of risk factors for carotid plaques

	OR	95% CI	*p* value
Age	1.10	1.03–1.20	0.006
10-Year cardiovascular risk^a^	1.15	0.89–1.64	0.384
Male gender	0.50	0.05–3.13	0.498
SLE	9.16	1.82–77.97	0.017

Analysis was performed on 79 subjects

^a^10-year cardiovascular risk was calculated using the Framingham equation

*CI* confidence interval, *OR* odds ratio, *SLE* systemic lupus erythematosus

### HS-cTNT is associated with carotid plaque in SLE patients

**Fig. 1:**
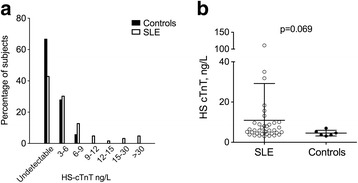
High-sensitivity cardiac troponin T (*HS-cTnT*) in systemic lupus erythematosus (*SLE*) patients. **a** Histogram showing the distribution of HS-cTnT in the serum of SLE patients (*white bars*) and controls (*black bars*). **b** Level of detectable HS-cTnT in serum is higher in SLE patients (*white rounds*) as compared to controls (*black rounds*). HS-cTnT was detectable in 37 SLE patients and in 6 controls. Undetectable HS-cTnT serum level <3 ng/L. The horizontal line is the mean and the whiskers are the SD

Systematic measurement of HS-cTnT in the serum revealed that HS-cTnT was detectable (i.e. >3 ng/L) in 37 (58.7%) SLE patients but only in 6 (33.3%) controls (*p* = 0.057). In addition, when detectable, the level of HS-cTnT in the serum was higher in SLE patients (median 5.87 (3–108.1) ng/L) as compared to controls (4.18 (3.12–6.99) ng/L; *p* = 0.069) (Fig. 1).

While comparing SLE patients with (*n* = 23) or without (*n* = 40) carotid plaques, we observed that older age (44 (30–63) vs 36 (29–59) years old, respectively; *p* < 0.001), a higher Framingham score (1.5 (1–18) vs 1 (1–9), respectively; *p* = 0.002), being overweight (65.2% vs 22.5%, respectively; *p* < 0.001), and a longer exposure to steroids (10 (0–21) vs 6.5 (0–16) years, respectively; *p* = 0.075) were associated with carotid plaques. Besides these well-known risk factors for atherosclerosis in SLE patients, detectable HS-cTnT was also associated with carotid plaques. Indeed, 87% of SLE patients with carotid plaques, against 42.5% of SLE patients without plaques (*p* < 0.001), had a detectable level of HS-cTnT in the serum (Table 3). Conversely, 20 (20/37, 54.1%) SLE patients with a detectable HS-cTnT, against 3 (3/26, 11.5%) with an undetectable HS-cTnT, had a carotid plaque (*p* < 0.001). In the multivariate analysis (Table 4), only BMI (*p* = 0.006) and HS-cTnT (*p* = 0.033) were statistically associated with carotid plaques in SLE patients. Overall, the risk of having a carotid plaque was increased by 9 (OR 9.26, 95% confidence interval 1.55–90.07) in SLE patients in whom HS-cTnT was detectable in the serum.

**Table 3:** Factors associated with carotid plaques in SLE patients

	No carotid plaque (*n* = 40)	Carotid plaque (*n* = 23)	*p* value
Age, years	36 (29–59)	44 (30–63)	<0.001
Female gender, *n* (%)	34 (85)	18 (78.3)	0.497
10-Year cardiovascular risk^a^, %	1 (1–9)	1.5 (1–18)	0.002
Overweight^b^	9 (22.5)	15 (65.2)	<0.001
eGFR^c^, ml/min/1.73 m^2^	96 (56–127)	76 (54–122)	0.294
HbA1c, %	5.4 (4.4–6.7)	5.5 (5–6.4)	0.336
Detectable HS-cTnT^d^	17 (42.5)	20 (87)	<0.001
Proteinuria/creatininuria, mg/mmol	18.7 (5–558)	22.7 (5–234)	0.425
Duration of SLE disease, years	11 (1–34)	14 (4–37)	0.138
SELENA-SLEDAI score	2 (0–13)	2 (0–8)	0.593
Lupus nephritis^e^, *n* (%)	19 (47.5)	14 (60.8)	0.306
AP antibodies^f^, *n* (%)	13 (32.5)	9 (39.1)	0.595
APS, *n* (%)	4 (10)	4 (17.4)	0.396
Cumulative years of steroid treatment, years	6.5 (0–16)	10 (0–21)	0.075
Hormonal contraception^g^, *n* (%)	12/34 (35.3)	3/18 (16.7)	0.158
Hydroxychloroquine, *n* (%)	40 (100)	22 (95/6)	0.184
Immunosuppressive therapy^h^, *n* (%)	28 (70)	19 (82.6)	0.421

Results are shown as median (range) or number (percentage)

Analysis was performed on 63 subjects

^a^10-year cardiovascular risk was calculated using the Framingham equation

^b^Overweight was defined as a body mass index >25 kg/m^2^

^c^Estimated glomerular filtration rate (eGRF) was calculated with the Modification of Diet in Renal Disease (MDRD) equation

^d^Detectable HS-cTnT referred to a high-sensitivity cardiac troponin T (HS-cTnT) serum level >3 ng/L

^e^Lupus nephritis was class III or class IV

^f^Antiphospholipid (AP) antibodies included lupus anticoagulant, anti-cardiolipin, or β2-glycoprotein 1 antibodies

^g^Hormonal contraception was progestin-only pill in all cases

^h^Immunosuppressive drugs included cyclophosphamide, azathioprine, mycophenolate mofetil, methotrexate, or rituximab

*APS* antiphospholipid syndrome, *HbA1c* haemoglobin A1c, *SELENA* Safety of Estrogens in Lupus Erythematosus National Assessment, *SLE* systemic lupus erythematosus, *SLEDAI* Systemic Lupus Erythematosus Disease Activity Index

**Table 4:** Multivariate analysis of risk factors for carotid plaques in SLE patients

	OR	95% CI	*p* value
10-Year cardiovascular risk^a^	1.30	0.99–2.07	0.168
BMI	1.35	1.13–1.74	0.006
Years of steroid treatment	1.09	0.99–1.24	0.110
Detectable HS-cTnT^b^	9.26	1.55–90.07	0.033
eGFR	1.01	0.99–1.03	0.513

Analysis was performed on 63 subjects

^a^10-year cardiovascular risk was calculated using the Framingham equation

^b^Detectable HS-cTnT referred to a high-sensitivity cardiac troponin T (HS-cTnT) serum level >3 ng/L

*BMI* body mass index, *CI* confidence interval, *eGRF* estimated glomerular rate filtration, *OR* odds ratio, *SLE* systemic lupus erythematosus

To further characterise the link between HS-cTnT and atherosclerosis in SLE patients, we analysed the relationship between detectable HS-cTnT and carotid IMT measurement on a longitudinal basis. A second vascular assessment was performed in 16 SLE patients 30 (16–44) months after the first evaluation. In 9 patients, the IMT increased by 0.03 (0.01–0.07) mm. Among these 9, 7 (77.8%) had a detectable HS-cTnT ranging from 3.21 to 8.94 ng/L. On the other hand, HS-cTnT was undetectable in the serum of 5 of the 7 (71.4%) SLE patients in whom IMT did not increase over time (*p* = 0.049). In addition, HS-cTnT measurements at baseline tended to correlate with IMT progression over time (Pearson *r* = 0.4, 95% CI –0.02 to 0.79; *p* = 0.062). Eventually, myocardial infarction occurred between 17 and 26 months after HS-cTnT measurement in 3 SLE patients in our series, who all had detectable HS-cTnT at baseline.

## Discussion

Prediction models such as the Framingham equation based on traditional cardiovascular risk factors are less accurate at identifying cardiovascular risks in SLE patients as compared to the general population. In our study, we show that low concentrations of cTnT, measured with a highly sensitive assay, are independently associated with subclinical atherosclerosis in SLE patients at apparent low risk for CVD according to the classic cardiovascular risk factors.

Detectable cTnT levels as measured by a highly sensitive assay were detectable in the majority of SLE patients in this cohort and in nearly 90% of SLE patients with carotid plaques, independent of traditional risk factors. These results should be considered in the context of prior findings with other biomarkers for stratifying cardiovascular risk in SLE patients. Recently, a combination of plasma biomarkers (including proinflammatory high-density lipoprotein, leptin, and TWEAK) and traditional risk factors (age and diabetes) was shown to have a better predictive capacity for the presence of an atherosclerotic plaque than a panel of traditional cardiac risk factors [16]. In our study, we showed that a single biomarker may be adequate for predicting the risk of accelerated atherosclerosis in SLE patients. Conversely, undetectable concentrations of HS-cTNT had a high negative predictive value (88%) for the diagnosis of subclinical atherosclerosis in our series, which is noteworthy because undetectable levels of this biomarker may prevent expensive and useless workup.

Detectable HS-cTnT was previously shown to be associated with coronary heart disease, mortality, and hospitalization for heart failure in individuals from a general population without known coronary heart disease and stroke [9]. In a population-based cohort of Dallas County, the prevalence of detectable HS-cTnT was 25% in 3546 individuals aged 30 to 65 years [17]. Interestingly, the frequency of detectable HS-cTnT in our study (58.7%) was close to the prevalence observed in patients older than 60 years [17, 18]. In the same vein, 4.8% of SLE patients in our study had a HS-cTnT level ≥30 ng/L as compared to less than 1% of the general population [19]. Such a percentage (>4% of patients with HS-cTnT level ≥30 ng/L) was actually observed in patients older than 60 years [20]. Eventually, a HS-cTnT level ≥10 ng/L was found in 24 subjects (2.2%) with the highest value of 20 ng/L in a work site-based population of 1072 middle-aged males (mean age 44 years) without any history or presence of CVD [21]. Comparing to our series of SLE patients (where >80% were females with a median age of 39 years), HS-cTnT levels ≥10 ng/L were found in 7 subjects (11.1%) with the highest value of 111 ng/L.

Our study was not designed to delineate the cause for detectable levels of HS-cTnT in SLE. Prior evidence has shown that exercise-induced cardiac ischaemia can lead to transient very low level increases in cardiac troponin levels as measured by a highly sensitive troponin I assay [22]. Thus, a continuous, very slow release of troponins from the myocardium might reflect ongoing cardiac myocyte cell death. Several other putative causes exist, however, for elevated cardiac troponin levels, including cardiopulmonary disease and chronic renal insufficiency [23–25]. Interestingly in our series, the percentage of SLE patients with past lupus nephritis were higher (64% vs 35%; *p* = 0.039) and the eGRF was lower (72 (54–122) vs 105 (63–127) ml/min/1.73 m^2^; *p* = 0.026) in patients with detectable HS-cTnT as compared to those with undetectable HS-cTNT. Moreover, although detectable HS-cTnT in our series was associated with subclinical atherosclerosis independently of classic cardiovascular risk factors, SLE patients with detectable HS-cTnT were older (42 (30–63) vs 33 (29–46) years old, *p* = 0.001) with a higher percentage of smoking habits (48% vs 23%; *p* = 0.039) and high blood pressure (15% vs 1%; *p* < 0.001) as reported in the general population [17, 20, 26, 27]. Hence, there may be more than one mechanism causing elevated troponin levels in SLE patients.

Our study should be interpreted within its limitations. First, our results were obtained with a limited number of consecutive SLE patients and controls. Larger studies are thus needed to confirm these preliminary results. Second, longitudinal changes in HS-cTnT concentrations were not assessed. Third, and more importantly, we did not have the power to demonstrate that HS-cTnT was actually associated with coronary heart disease. It should, however, be noticed that a carotid plaque is: 1) direct evidence of atherosclerosis (whereas carotid IMT measurement is only a surrogate marker) [28]; and 2) associated with cardiovascular events in SLE patients [29–31]. Eventually, myocardial infarction occurred between 17 and 26 months after HS-cTnT measurement in 3 SLE patients who all had both detectable HS-cTnT and carotid plaques. However, the clinical importance of monitoring HS-cTnT levels for risk of progression to symptomatic CVD is still to be determined. Further studies are also needed to assess whether therapies such as statin and/or aspirin could be considered for SLE patients re-classified at higher risk for CVD according to HS-cTnT.

## Conclusion

A detectable HS-cTnT concentration is independently associated with subclinical atherosclerosis in asymptomatic SLE patients at apparent low risk for CVD according to traditional risk factors. These results raise the possibility that this easily obtained biomarker is useful for more rigorous risk stratification and primary prevention of CVD in SLE patients. Future studies with larger cohorts are now warranted to assess the merit of this biomarker to predict the further occurrence of cardiovascular events and help a future preventive treatment strategy.

## Abbreviations

AP: Antiphospholipid
APS: Antiphospholipid syndrome
BMI: Body mass index
CI: Confidence interval
CV: Coefficient of variation
CVD: Cardiovascular disease
ECG: Eletrocardiography
eGRF: Estimated glomerular rate filtration
HS-cTnT: High-sensitivity cardiac troponin T
IMT: Intima media thickness
OR: Odds ratio
SELENA: Safety of Estrogens in Lupus Erythematosus National Assessment
SLE: Systemic lupus erythematous
SLEDAI: Systemic Lupus Erythematosus Disease Activity Index
